# Forecasting Carbon Price in China: A Multimodel Comparison

**DOI:** 10.3390/ijerph19106217

**Published:** 2022-05-20

**Authors:** Houjian Li, Xinya Huang, Deheng Zhou, Andi Cao, Mengying Su, Yufeng Wang, Lili Guo

**Affiliations:** 1 College of Economics, Sichuan Agricultural University, Chengdu 611130, China; 14159@sicau.edu.cn (H.L.); huangxinya@stu.sicau.edu.cn (X.H.); zhoudeheng@stu.sicau.edu.cn (D.Z.); caoandi@stu.sicau.edu.cn (A.C.); 13487@sicau.edu.cn (Y.W.); 2 College of Economics, Guangxi University for Nationalities, Nanning 530006, China

**Keywords:** multivariate long short-term memory, multilayer perceptron, support vector regression, recurrent neural network, carbon price forecasting

## Abstract

With the global concern for carbon dioxide, the carbon emission trading market is becoming more and more important. An accurate forecast of carbon price plays a significant role in understanding the dynamics of the carbon trading market and achieving national emission reduction targets. Carbon prices are influenced by many factors, which makes carbon price forecasting a complicated problem. In recent years, deep learning models are widely used in price forecasting, because they have high forecasting accuracy when dealing with nonlinear time series data. In this paper, Multivariate Long Short-Term Memory (LSTM) in deep learning is used to forecast carbon prices in China, which takes into account the factors affecting the carbon price. The historical time series data of carbon prices in Hubei (HBEA) and Guangdong (GDEA) and three traditional energy prices affecting carbon prices from 5 May 2014 to 22 July 2021 are collected to form two data sets. To prove the forecast effect of our model, this paper not only uses Multivariate LSTM, Multilayer Perceptron (MLP), Support Vector Regression (SVR), and Recurrent Neural Network (RNN) to forecast the same data, but also compares the forecast results of Multivariate LSTM with the existing research on HBEA and GDEA forecast based on deep learning recently. The results show that the MAE, MSE, and RMSE obtained by the Multivariate LSTM are all smaller than other prediction models, which proves that the model is more suitable for carbon price forecast and offers a new approach to carbon prices forecast. This research conclusion also provides some policy implications.

## 1. Introduction

With the rapid economic growth of all countries, the greenhouse gases such as carbon dioxide produced by human beings in the process of production and living have increased dramatically, resulting in sustained global warming. Global warming has affected all aspects of human activities to varying degrees. In order to cope with global warming effectively, the Kyoto Protocol adopted in 1997 regards the carbon emission trading market as a new pathway to control greenhouse gas emissions. As the largest developing country in the world, China’s rapid economic growth is accompanied by huge energy consumption, resulting in China’s greenhouse gas emissions accounting for a large proportion of the global total. Therefore, China has great potential for carbon emission reduction, and it is extremely urgent to establish a carbon emission trading market. Since 2013, China has carried out seven carbon emission trading pilots in two provinces and five cities, namely Hubei, Guangdong, Shenzhen, Shanghai, Beijing, Tianjin, and Chongqing, which kicked off China’s carbon emissions trading market from scratch. With the acceleration of carbon emission reduction, China’s carbon trading market is becoming more and more mature. The national unified carbon emission trading market was formally opened on 16 July 2021, which marked that China’s carbon trading market moved from a decentralized pilot to national unification. As a result, the relevant research on the domestic carbon emission trading market is of great practical significance. It is not only conducive to the in-depth development of China’s low-carbon economy but also provides reference suggestions for the construction and improvement of carbon trading markets around the world.

With the continuous in-depth growth of the carbon trading market, the financial trend of the market is becoming increasingly significant. The carbon market not only has the general attributes of other financial markets but also has certain particularities and externalities. In the carbon trading market, carbon prices are the most concerned core element, because the level and fluctuation of carbon prices can directly reflect the market performance. Accurate forecasting of carbon prices is not only beneficial to the comprehensive, sustainable and healthy development of the carbon market but also conducive to better planning and decision-making by the government and market participants [1]. Although scholars have made some achievements in the research of carbon price prediction, there are still some problems to be solved. Meanwhile, as the carbon emission trading market in China started relatively late, the research on carbon price prediction is still very limited. Therefore, the effective method to predict carbon price in China deserves further study.

In the existing literature, most carbon price prediction is mainly based on the time series data of carbon price itself. However, a large number of studies have proved that carbon price is influenced by many factors, such as policy factors [2], weather conditions [3], and the energy market [4]. It is worth noting that the main cause of greenhouse gas production is still the burning of fossil energy. Among many influencing factors, energy price is regarded as the core determinant of carbon price [5]. Hao and Tian [6] pointed out that the carbon price forecast based on multi-factors can provide more valuable information for the carbon trading market because it considers and analyzes the influence of exogenous variables. However, Zhu et al. [7] believed that although the multi-factor carbon price prediction model could consider the influence of exogenous variables, there might be accumulated errors in the prediction process, which would lead to the failure of carbon price prediction. Therefore, it is a challenging task to predict carbon prices by multi-factors. To solve this challenging task, this paper introduces the Multivariate Long Short-Term Memory (LSTM) model in deep learning into carbon price prediction and takes both energy price and carbon price as the input characteristics of the prediction model. The reasons for choosing LSTM are as follows: On the one hand, LSTM does not need feature engineering in the construction process, and can well mine the time correlation between nonlinear and non-stationary time series data. On the other hand, LSTM has been used in electricity price forecast [8], crude oil price forecast [9], stock price forecast [10], and other aspects that have achieved good prediction results.

Compared with the existing research, the main innovations and contributions of this study are as follows: Firstly, previous studies are based on historical time-series data of carbon price itself to forecast carbon prices, while ignoring the importance of influencing factors in carbon price prediction. The carbon emissions trading market in China is relatively complex, and the fluctuation of carbon prices is affected by various factors, so the historical time series data of carbon price itself cannot summarize all the information. Therefore, considering that carbon price is influenced by many factors, this paper takes energy price as the most important influencing factor and carbon price as input variables at the same time, thereby improving the authenticity and accuracy of the forecast [11]. Secondly, to enrich the existing carbon price forecasting research and explore the effectiveness and practicability of deep learning in carbon price forecasting, the Multivariate LSTM is creatively introduced into carbon price forecasting. Compared with MLP, SVR, RNN, and other existing studies, the superiority of our model in carbon price prediction is confirmed. Finally, previous studies are mostly based on EU carbon prices, and there are fewer studies on the carbon price in China. According to the active degree of carbon trading, carbon markets in Hubei and Guangdong are taken as research objects, which enriches the related research of the carbon market in China. Meanwhile, the forecasting of carbon prices in the two pilots is of great significance for understanding the dynamics of the carbon market in China, further building a unified carbon market in China, and achieving the national emission reduction targets.

The rest of this paper is structured as follows: Section 2 gives a detailed overview of the related literature on prediction models. RNN and LSTM model is introduced in Section 3. Data selection and preprocessing are explained in Section 4. The experimental results are presented in Section 5. The LSTM prediction results are discussed with the existing research in Section 6. Section 7 sums up the whole paper and proposes policy implications.

## 2. Literature Review

In the existing literature, researchers have made a lot of predictions in different fields, including finance [12], tourism demand [13], carbon price [14], and so on. Scholars have put forward a large number of forecasting models to improve the effectiveness and accuracy of forecasting. This section will provide a detailed literature review from the perspective of forecasting models.

Early scholars mainly adopted autoregressive (AR), generalized autoregressive conditional heteroscedasticity (GARCH), etc. Early scholars mainly used the econometric models such as Autoregressive (AR), Generalized Autoregressive Conditional Heteroscedasticity (GARCH), and Autoregressive Moving Average (ARMA) to make predictions. Byun and Cho [15] used GARCH, Implied Volatility models, and the k-nearest Neighbor Algorithm to, respectively, predict the carbon price and revealed that GARCH was the most effective forecasting technique. Paolella and Taschini [16] demonstrated that the mixture GARCH models were more effective than the normal GARCH models. Torres et al. [17] used the ARMA model to predict the wind speed in five places with different topographic features. Tang et al. [18] used two mixed models ARMA-GARCH and AR-GARCH to forecast the stock price and the results showed that the ARMA-GARCH model had a better forecasting effect. Although these conventional econometric models can obtain effective prediction results, they are based on the assumption that the data is stable and linear, so they are not capable of capturing the nonlinear characteristics of time series data [19].

For overcoming this problem, scholars have introduced machine learning technology. There is plenty of literature to prove that machine learning models are more suitable for dealing with non-stationary and nonlinear carbon price sequences than traditional econometric models. Huo et al. [20] used a support vector machine (SVM) and random forest regression (RFR) to forecast the electrical load of different cities, and the results proved that the two machine learning models were both better choices for power load forecasting. Wang et al. [21] predicted the price and volume of carbon trading in Beijing by using Back Propagation (BP). Roos et al. [22] introduced the dynamic Bayesian network method into the short-term passenger flow prediction. In addition, some scholars demonstrated that the hybrid model combining machine learning model and econometric model can further improve the prediction performance of the model. Shi et al. [23] put forward a hybrid model of ARMA, backpropagation neural network (BPNN), and Markov to predict the stock prices and achieved good prediction results. Zhu and Wei [24] used the combination of Autoregressive Integrated Moving Average (ARIMA) and Least Squares Support Vector Machine (LSSVM) to forecast the EU ETS carbon price and proved that the ARIMA-LSSVM model was superior to traditional models in forecasting accuracy. Sun et al. [25] used variational pattern decomposition (VMD) to decompose the carbon price, and then used a spike neural network (SNN) to predict the carbon price. The experimental results show that the VMD-SNN model has a good prediction effect. However, the above models have not broken through their limitations. The learning ability of conventional machine learning models with highly complex characteristics will be restricted when dealing with multi-dimensional time series.

In recent years, deep learning models have become the most popular research method because of their better prediction accuracy and reliability, and to a certain extent, it solves the limitations of conventional machine learning. Wang et al. [26] used traditional machine learning and deep learning to predict four different data sets which showed that deep learning had better prediction ability. The deep learning method has been successfully applied in many forecasting fields, such as wind power forecasting [27], power load forecasting [28], and carbon price forecasting [29]. In addition, the recurrent neural network model (RNN) in deep learning is very effective in fitting time-series data. As a special RNN model, LSTM is widely used in various fields. For example, Nelson et al. [30] constructed the LSTM model to predict the future trend of stock price based on the historical time series data of stock price, which confirmed the effectiveness of the LSTM model in stock price prediction. Altché and de La Fortelle [31] used LSTM in highway trajectory prediction and proved that this model can accurately predict the future trajectory of vehicles on highways. Cen and Wang [32] predicted the prices of West Texas Intermediate crude oil and Brent crude oil by LSTM, and the results showed that the model had better prediction accuracy. By combing the literature, it can be found that the research on carbon price prediction based on deep learning is still very limited. Therefore, applying LSTM to carbon price prediction can not only enrich the existing research on carbon price prediction but also verify the effectiveness of deep learning in the field of the carbon price.

## 3. Methodology

### 3.1. RNN and LSTM Model

**Figure 1:**
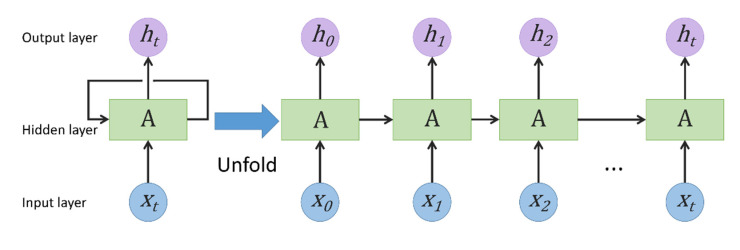
Structure of Recurrent neural network. $h_{t}$ and $x_{t}$ represent the output and input at time t, respectively.

RNN is a mainstream deep learning algorithm, which was proposed by Rumelhart et al. as early as 1986 [33]. Compared with the conventional neural network models, RNN has special memory functions, which is suitable for dealing with sequence-related problems such as various time-series learning, language modeling, image processing, etc. RNN through the mutual communication between hidden layers stores the previous output results and takes them to the next hidden layer for training. A fundamental structure of RNN is depicted in Figure 1 Theoretically, the parameter sharing mechanism and cyclic feedback mechanism can enable RNN to process arbitrary time series data. However, in the actual training process, the conventional RNN usually faces the problems of gradient disappearance and explosion, and cannot obtain long-term dependency information, so it cannot process long time series data. Therefore, to solve the long-term dependence of RNN, many scholars have proposed some improved methods, such as Gated Recurrent Units (GRU) and LSTM, etc.

**Figure 2:**
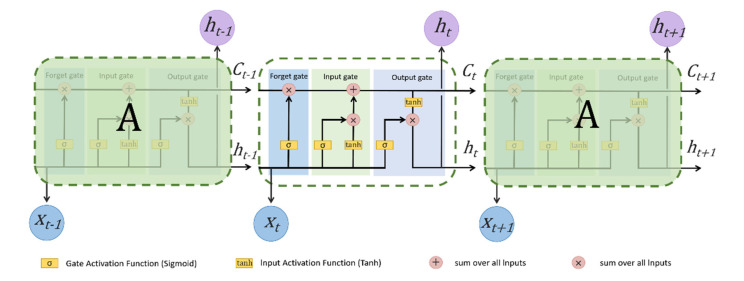
Structure of long short-term memory. $h_{t}$ , $C_{t}$, and $x_{t}$ represent the output, cell state, and input at time t, respectively.

Among them, LSTM was proposed by Hochreiter and Schmidhuber in 1997 [34] and improved and extended by Alex Graves in 2010. LSTM, as a variant of RNN, introduces addition operation into the network through gate control, thus solving the problems of gradient disappearance and explosion. In other words, LSTM can learn long-term dependency information more effectively than RNN. Therefore, LSTM with long-term memory capability has a strong advantage in processing and predicting highly correlated time series data. The structure of LSTM mainly adds three control gates based on RNN: input gate, output gate, and forgetting gate. A fundamental structure of LSTM is depicted in Figure 2.

The LSTM operation process needs the following four steps:

Step 1: To decide which information in the previous cell state $C_{t-1}$ should be removed or retained. This task is realized by the forget gate, it takes input as the previous output $h_{t-1}$ and the present input vector $x_{t}$, and then applies the sigmoid function to the forget gate. The forget gate output is $f_{t}$, which is assigned to $C_{t-1}$. The computation equation is as follows:

(1) $$f_{t}=\sigma \left(W_{f}[h_{t-1},x_{t}\right]+b_{f})$$

Step 2: To decide which information in the present cell state $C_{t}$ should be stored. This task is accomplished in two steps, first through the input gate to decide what information should be updated and then through the tanh layer to obtain a candidate vector value $C_{t}^{1}$ to the state. The computation equations are as follows:

(2) $$i_{t}=\sigma \left(W_{i}[h_{t-1},x_{t}\right]+b_{i})$$

(3) $$C_{t}^{1}=\tan h\left(W_{c}[h_{t-1},x_{t}\right]+b_{c})$$

Step 3: To convert the previous cell state $C_{t-1}$ into the present cell state $C_{t}$. The information that was previously decided to be removed is discarded by multiplying $C_{t-1}$ by the forget gate output $f_{t}$, and then the candidate vector value $C_{t}^{1}$ is added which needs to be scaled according to the input gate output $i_{t}$. The computation equation is as follows:

(4) $$C_{t}=f_{t}*C_{t-1}+i_{t}*C_{t}^{1}$$

Step 4: To decide the output values through the output gate. The sigmoid layer determines which information of $C_{t}$ needs to be output. In the last stage, the result of the processing cell state of the tanh layer is multiplied by the output of the Sigmoid layer. The computation equations are as follows:

(5) $$O_{t}=\sigma \left(W_{O}[h_{t-1},x_{t}\right]+b_{O})$$

(6) $$h_{t}=O_{t}*\tan h\left(C_{t}\right)$$

In these equations, σ is the activation function named Sigmoid, t is the present time state, $W_{f}$, $W_{i}$, $W_{c}$ and $W_{o}$ represent the corresponding weight vectors, and $b_{f}$, $b_{i}$, $b_{c}$ and $b_{o}$ represent the corresponding deviation vectors.

### 3.2. Model Evaluation Index

In order to assess the forecast accuracy of the model, according to the actual prices and the predicted prices of the carbon emission trading market, this paper selects Mean Absolute Error (MAE), Mean Squared Error (MSE), and Root-Mean-Square Error (RMSE) as the evaluation indexes of the model. MAE refers to the average value of absolute deviations among predicted and actual values, which can well reflect the real error of the predicted value. MSE refers to the average value of square deviation between the predicted values and the actual values, which can be used to evaluate the change of the function. RMSE refers to the square root of MSE, which is used to calculate the deviation between the predicted value and the actual value. The computation equations are as follows:

(7) $$\mathrm{MAE}=\frac{1}{N}\sum_{i=1}^{N}\left|y_{i}-p_{i}\right|$$

(8) $$\mathrm{MSE}=\frac{1}{N}\overset{N}{\underset{i=1}{\sum }}{\left(y_{i}-p_{i}\right)}^{2}$$

(9) $$\mathrm{RMSE}=\sqrt{\frac{1}{N}\overset{N}{\underset{i=1}{\sum }}{\left(y_{i}-p_{i}\right)}^{2}}$$

In these equations, $y_{i}$ is the predicted value, $p_{i}$ is the actual value, and N is the sample quantity. Generally, the smaller the values of MAE, MSE, and RMSE, the smaller the error between the predicted carbon price and the actual carbon price, indicating that the prediction performance of the model is better.

## 4. Data Description

The carbon price is impacted by many factors, which makes carbon price forecasting a very complicated problem. The main source of carbon emissions is still the combustion of fossil energy, so this paper considers the energy price that affects the carbon price. Meanwhile, the time-series data of carbon prices have autocorrelation, when forecasting the carbon price, we can predict its future price according to the historical time series data of carbon price itself. As a result, this paper takes carbon price and energy price as input features to predict future carbon prices.

### 4.1. Carbon Emission Trading Price Data

Carbon emission trading pilots in China started late, and there are some differences among these carbon trading pilots. Table 1 shows the trading situation of seven carbon trading pilots—Hubei, Shenzhen, Guangdong, Beijing, Shanghai, Tianjin, and Chongqing, from 5 May 2014 to 22 July 2021. In Table 1, the carbon trading markets in Hubei, Shenzhen, and Guangdong are the most active, and their trading days have reached more than 1500 days in the selected time range. The Hubei carbon trading market is the only carbon trading market located in the middle of China. The Hubei carbon trading market has been widely concerned since its opening in 2014, and the industrial development level of Hubei province is close to the national average level. As a result, there has always been a saying in the industry that “When Hubei becomes successful, China becomes successful”. The Guangdong carbon market is the first provincial carbon trading market in China, and it is developing steadily. Guangdong province is the largest production and trade province with the highest GDP in China, and its economic development is in a leading position in China. Therefore, the experience of Guangdong province can be used as a development sample for other regions or provinces. However, the Shenzhen carbon market will propose a new trading product with a year every year and implement differential pricing for them. The new trading product involves transactions in the corresponding year and subsequent years, which leads to several different products in the Shenzhen carbon market at the same time. Therefore, it is difficult to determine the time series data of carbon prices in Shenzhen.

To sum up, this paper takes the Hubei carbon market (abbreviation: HBEA) and the Guangdong carbon market (abbreviation: GDEA) as research objects. The daily closing prices of HBEA and GDEA are selected as experimental data, the period of which is from 5 May 2014 to 22 July 2021, excluding the daily data with the trading volume of 0. All data used are gathered from the Wind database.

### 4.2. Energy Price Data

Fossil energy prices mainly affect carbon prices through two paths. One path is that the energy prices directly affect the carbon prices by affecting the energy consumption. As the energy consumption depends on the energy prices to a great extent, when the energy prices decrease, the emission control enterprises will be more inclined to use fossil energy, which will lead to an increase in carbon emissions and eventually changes in carbon prices. Conversely, when the energy price rises, enterprises will choose other energy sources and reduce the use of fossil energy which will affect their carbon emissions. Another path is that the energy prices have an indirect impact on the carbon price by influencing the energy utilization rate. That is, when energy prices rise, in order to control the cost, enterprises will develop new technologies to improve the utilization rate of energy, which will affect the carbon emissions and ultimately the carbon price. Therefore, changes in crude oil, natural gas, and coal prices will cause carbon price changes.

Firstly, the crude oil price is an important factor affecting the global macro-economy [35], and China’s economic and social development can not be separated from the support of crude oil. Since China relies too much on imported crude oil, Brent crude oil futures price is selected as the representative of oil price in this paper. Currently, Brent crude oil futures price in Beihai is one of the most important pricing benchmarks in the world. According to the data published by London Intercontinental Exchange (ICE), this price is used as the pricing benchmark by more than two-thirds of the world’s international oil trade. Secondly, as a low-carbon, clean and efficient fossil energy, natural gas is considered to be the leading force in the green transformation of energy. If the electric power industry uses natural gas instead of coal, it can greatly reduce carbon emissions. As there is no authoritative natural gas price index in China at present, this paper selects the New York Mercantile Exchange (NYMEX) natural gas price as the representative of natural gas price. Since the listing of the NYMEX natural gas futures contract in 1990, the trading volume and positions have been increasing. NYMEX natural gas futures contract price is also widely used as the benchmark price of natural gas. Finally, in China, a big industrial country, coal is the main primary energy and plays a key role in the national economy. The coal price is considered the core determinant of carbon price [36]. As China is a big importer of coal, mainly imported from Australia [37], this paper selects the Newcastle coal spot price in Australia as the representative of coal price. Australia’s Newcastle port is the largest coal export port in the world while the Newcastle coal spot price is one of the important indicators to measure the international coal price. Table 2 shows the selection of energy prices, and all data are available from the Wind database.

### 4.3. Data Preprocessing

In this paper, three energy prices and carbon prices are taken as explanatory variables at the same time. Therefore, we need to sort the obtained original data and fill the missing values with the previous day’s prices, to obtain two complete time-series data sets. Since each time series data does not belong to the normal distribution, so it is impossible to fill the missing value with the average value. Table 3 shows the descriptive statistical results of two-time series data sets. It can be seen from Table 3 that the average daily prices of HBEA and GDEA are 23.830 and 22.537, respectively, whereas the average daily price of natural gas is less than 3, and the average daily prices of crude oil and coal are over 59, which means that the average values of various variables are quite different. In order to eliminate the differences among the variables, it is necessary to normalize all variables. The computation equation is as follows:

(10) $$X=\frac{x-\min}{\max-\min}$$

In this equation, min is the minimum value of each variable in the data set and max is the maximum value of each variable in the data set.

**Figure 3:**
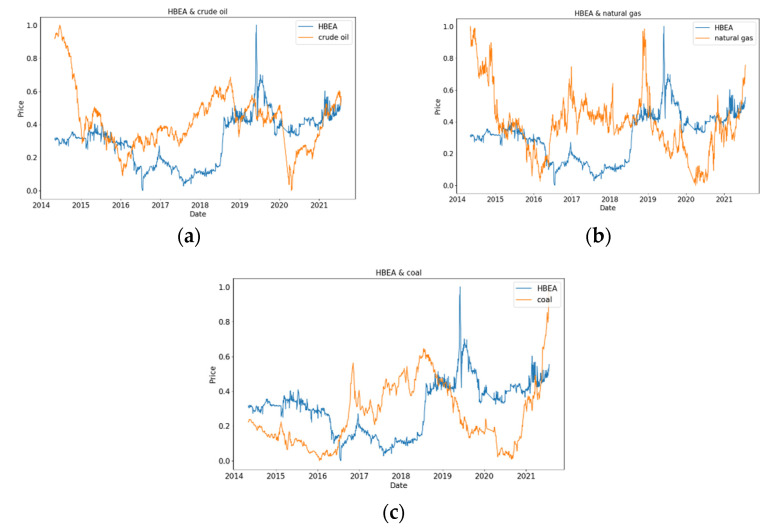
HBEA compared with the energy analysis. (**a**) HBEA and crude oil; (**b**) HBEA and natural gas; (**c**) HBEA and coal.

Figure 3 shows the comparison of normalized HBEA with crude oil, natural gas, and coal. In Figure 3a, the fluctuations of HBEA and crude oil are very similar in the selected time interval of the sample, which is a change process of first falling and then rising, then falling and then rising. However, HBEA increases sharply to its peak in May to June of 2019, whereas crude oil decreases sharply to its minimum from January to April 2020 to reach the minimum. In Figure 3b, HBEA and natural gas developed in opposite directions from June 2016 to 2020, and the trends of HBEA and natural gas were roughly the same in the remaining periods. In Figure 3c, from July 2018 to May 2019, the fluctuation of HBEA is relatively stable, whereas coal is gradually decreasing. In recent years, HBEA shows a gradual upward trend, whereas coal shows a sharp upward trend.

**Figure 4:**
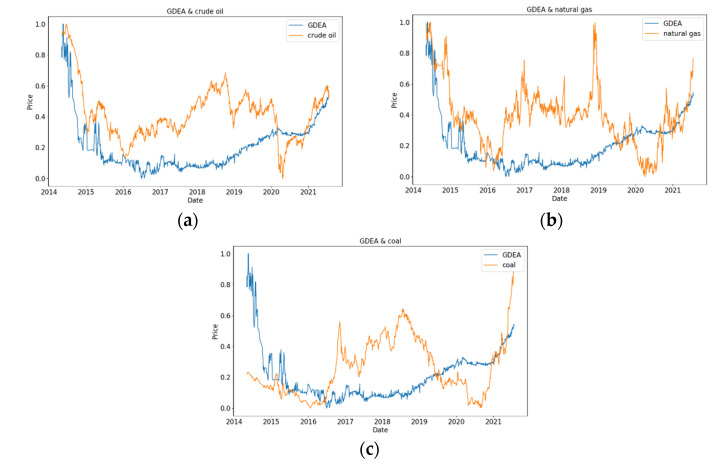
GDEA compared with the energy analysis. (**a**) GDEA and crude oil; (**b**) GDEA and natural gas; (**c**) GDEA and coal.

Figure 4 shows the comparison of normalized GDEA with crude oil, natural gas, and coal. At the same time, it can be seen that within the selected time range, GDEA will gradually decrease before 2017, and then GDEA will gradually increase. In Figure 4a, on the whole, the fluctuations of GDEA and crude oil are similar, indicating that there is an obvious positive correlation between them. In Figure 4b, it can be observed that natural gas has a downward trend from 2019 to 2020, whereas the values of GDEA show a gradual upward trend. In Figure 4c, there is an opposite relationship between GDEA and coal from 2019 to 2021.

**Figure 5:**
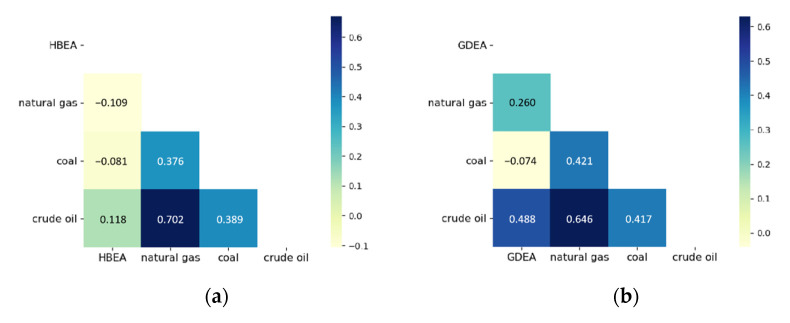
Pearson Heat Map. (**a**) HBEA; (**b**) GDEA.

The correlations between the four variables of the two data sets are shown in the Pearson Heat Map in Figure 5. As can be seen from Figure 5a, HBEA is positively correlated with crude oil, and the correlation coefficient is +0.118. While HBEA is negatively correlated with natural gas and coal, its correlation coefficients are −0.109 and −0.081, respectively. In Figure 5b, GDEA is positively correlated with crude oil and natural gas, and their correlation coefficients are +0.488 and +0.260, respectively, indicating that GDEA has a strong positive correlation with crude oil and natural gas. While GDEA is negatively correlated with NEWC.

## 5. Empirical Results

Accurately predicting the carbon price is of great significance for countries to achieve carbon emission reduction targets. The purpose of this experiment is to forecast Hubei carbon emission trading price (HBEA) and Guangdong carbon emission trading price (GDEA) by using Multivariate LSTM. The Multivariate LSTM model is trained with 300 epochs, the batch size is 32. The time window length is 10, that is, the data of the first 10 days are used to calculate the carbon price of the 11th day. This paper uses Multivariate LSTM, SVR, MLP, and RNN to forecast the same data, and the results are compared and analyzed to prove the prediction effect of our model.

### 5.1. Carbon Price Forecasting in Hubei Province

**Figure 6:**
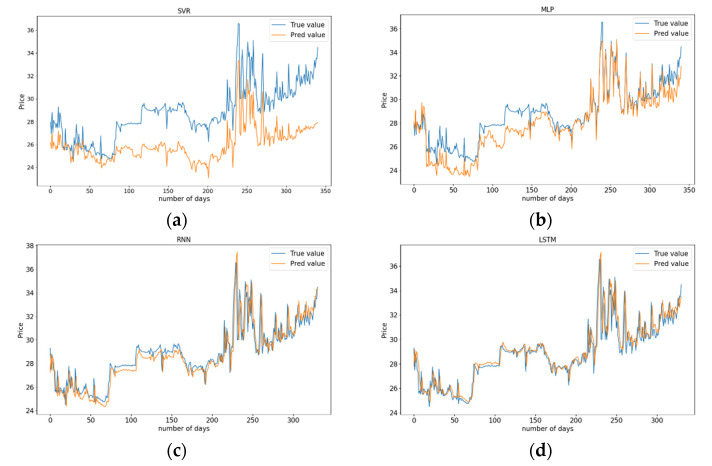
Models predicted and actual HBEA values. (**a**) SVR; (**b**) MLP; (**c**) RNN; (**d**) LSTM.

The four models use HBEA and three energy prices (crude oil, natural gas, and coal) to predict HBEA values from 5 May 2014 to 22 July 2021. There are 1704 observations in the data set, of which the previous 80% are used as the training set, and the remaining 20% are used as the test set to evaluate the prediction performance of the model. Figure 6 depicts the line plots of predicted HBEA values and actual HBEA values of the four models based on the test set. In Figure 6a, the prediction effect of SVR is not good. Although the predicted values are consistent with the situation of actual values, it only realizes the prediction of the HBEA trend. However, there are significant differences between the predicted values and the actual values, and the predicted values are generally lower than the real values. The actual and predicted HBEA values obtained by the MLP model are shown in Figure 6b. The predicted values of the MLP model are not close to the actual values in the first 180 days whereas the model performs better in the last part. The prediction result of the RNN model is shown in Figure 6c. The predicted values are very consistent with the actual values of HBEA in trend, but there is always a certain error between the predicted values and the actual values at each time point, which shows that RNN has obvious hysteresis. In Figure 6d, the predicted value of HBEA obtained by Multivariate LSTM has a high degree of fitting with the actual value.

In conclusion, through the comparison of the actual values and the predicted values of SVR, MLP, RNN, and Multivariate LSTM, it can be seen that the predicted prices obtained by Multivariate LSTM are more consistent with the actual prices of HBEA. Especially in the places where HBEA fluctuates drastically, the prediction effect of Multivariate LSTM is better than other prediction models.

To further prove the prediction effect of Multivariate LSTM, the evaluation indexes of each prediction model are calculated, and the results of the four models are shown in Table 4. It can be found from Table 4 that the prediction indexes of Multivariate LSTM are smaller than those of other models. From the MAE point of view, the error of LSTM is 77.77% less than SVR, 45.11% less than MLP, and 9.93% less than RNN. From the point of MSE, the prediction performance of Multivariate LSTM is 89.8% higher than SVR, 35.9% higher than MLP, and 14.48% higher than RNN. From the perspective of RMSE, the error of LSTM is 68.07% lower than SVR, 19.97% lower than MLP, and 7.56% lower than RNN. In a word, all the prediction indexes show that the prediction accuracy of Multivariate LSTM is superior to that of other prediction models, which indicates Multivariate LSTM is fitter for predicting the carbon emissions trading prices in Hubei.

### 5.2. Carbon Price Forecasting in Guangdong Province

**Figure 7:**
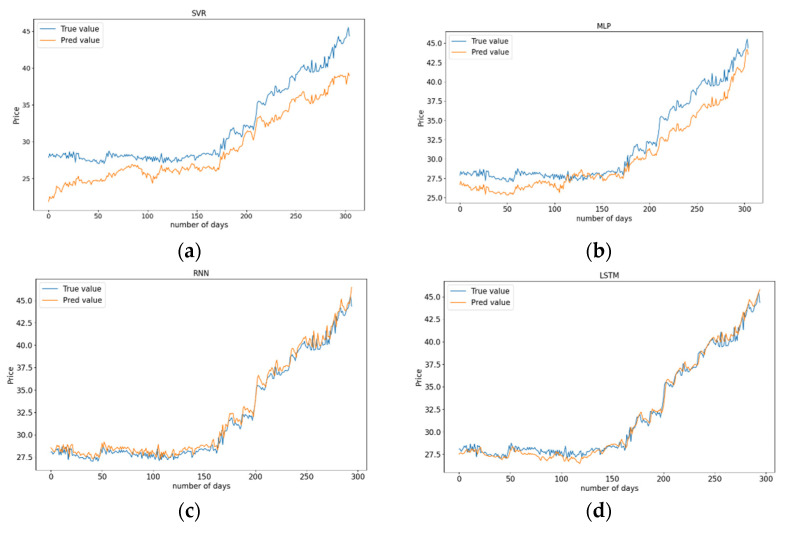
Models predicted and actual GDEA values. (**a**) SVR; (**b**) MLP; (**c**) RNN; (**d**) LSTM.

The four models use the time series data of GDEA, crude oil, natural gas, and coal from 5 May 2014 to 22 July 2021 to predict the GDEA values. The data set with 1520 observations are processed in the same way as HBEA. Figure 7 depicts line graphs of actual GDEA values and predicted GDEA values of the four models. In Figure 7a, it can be seen that the SVR model performs worst in forecasting GDEA prices. There is an obvious difference between the predicted values and the actual values, and the predicted values are always lower than the actual values. In Figure 7b, it can be seen that compared with SVR, MLP has a better performance prediction effect which can well predict the trend of GDEA, but there is still a big gap between the predicted values of MLP and the actual values of GDEA. The predicted result of RNN is shown in Figure 7c. The predicted GDEA values are very consistent with the actual GDEA values in trend. Although the predicted values are very close to the actual GDEA values, there is always a certain error between the predicted values and the actual values, which has a certain delay. In Figure 7d, for predicting Guangdong carbon emission trading prices, the predicted values curve obtained by LSTM can well approach the actual GDEA values. Similarly, compared with other models, the forecast accuracy of Multivariate LSTM is still better.

Table 5 shows the evaluation index values of four forecasting models, and all the indexes of Multivariate LSTM are superior to other forecasting models. From the perspective of MAE, the error obtained by LSTM is 85.23% smaller than SVR, 37.67% smaller than MLP, and 22.77% smaller than RNN. From the MSE point of view, the prediction performance of LSTM is 96.79% higher than SVR, 62.96% higher than MLP, and 31.38% higher than RNN. From the perspective of RMSE, the error of LSTM is 82.09% lower than SVR, 39.15% lower than MLP, and 17.15% lower than RNN. In a word, LSTM and RNN are better than SVR and MLP in forecasting the carbon trading prices in Guangdong, and Multivariate LSTM has the best prediction effect. Therefore, Multivariate LSTM is more suitable for forecasting the carbon emissions trading price in Guangdong.

## 6. Discussion

Recently, some scholars have introduced machine learning and deep learning technology into carbon price forecasting. In the existing research, this paper selects four models as the benchmark models. The comparison between our model and these models based on RMSE values is shown in Table 6. These models are briefly summarized here. Sun and Huang [38] put forward a carbon price forecasting model based on EMD-VMD-LSTM. The RMSE obtained by this model is 1.04 in the process of carbon price prediction in Hubei. Wang et al. [39] used the VMD-SE-DRNN-GRU mixed model to predict the carbon price in Hubei. The forecasting process includes three parts, namely, feature extraction, forecasting, and integrated forecasting, and the RMSE of this model is 1.048. Xiong et al. [40] predicted the carbon price in Guangdong by using the VMD-FMRVR-MOWOA mixed model and proved that the model has a good prediction effect, with an RMSE of 0.57. The verification shows that EEMD-LDWPSO-wLSSVM is an effective method to predict carbon price. According to the research of Wang et al. [41], CEEMDAN-SE-LSTM- RF can predict the carbon price in Guangdong and achieve a good prediction effect, with an RMSE of 1.295. These hybrid models have made an important contribution to the prediction of the carbon price.

However, all the above studies have a common feature which is that the data they used in the modeling process is only the time series of the carbon price itself, without considering the related variables that affect the carbon price. It is the biggest difference from them that when we select the modeling input features, we not only consider the historical time series data of carbon prices but also the data of crude oil, natural gas, and coal prices. By comparing the RSME of our model with the above models, it can be concluded that the accuracy of the multivariate LSTM model considering the influencing factors in this paper is better than the selected mixed models.

## 7. Conclusions and Policy Implications

Carbon trading is considered to be an important method for reducing carbon emissions, and setting reasonable carbon prices is beneficial to the development of the carbon trading market. Accurate forecasting of the domestic carbon price is of great significance for enhancing China’s position in the global carbon emission trading market and promoting China’s carbon emission reduction targets. However, the carbon price is non-stationary and non-linearity and is affected by many factors, which make carbon price forecasting a challenging task. In recent years, deep learning models have been successfully applied to many complicated nonlinear sequence predictions. Therefore, the purpose of this paper is to forecast the carbon price in China’s carbon market based on using the Multivariate LSTM in deep learning.

Firstly, according to the active degree of carbon trading, we choose the carbon price of Hubei (HBEA) and Guangdong (GDEA) as the research objects. Considering that carbon price is affected by energy price, crude oil price, natural gas price, and coal price are identified as three important variables affecting carbon price. Five variable data from 5 May 2014 to 22 July 2021 are collected from the Wind database and processed into two complete data sets. There are 1704 observations in the Hubei data set and 1520 observations in the Guangdong data set.

Secondly, the carbon prices in Hubei and Guangdong were predicted by using the Multivariate LSTM. For comparison, this paper not only uses SVR, MLP, and RNN to forecast the same data but also compares the forecasting results of Multivariate LSTM with the existing research. When forecasting HBEA values, the Multivariate LSTM prediction results indicate that MAE is 0.617, MSE is 0.957, and RMSE is 0.978. Additionally, in the forecast of GDEA values, the prediction results of Multivariate LSTM show that MAE is 0.407, MSE is 0.293 and RMSE is 0.541. Compared with other models, the prediction accuracy of Multivariate LSTM is still better. In general, the Multivariate LSTM model is more suitable for carbon price prediction, which provides a new method for carbon price prediction.

Finally, based on the research results, the policy implications are put forward as follows: (1) Regulators of the carbon market should pay attention to the energy price closely related to the carbon price. In the development of the carbon trading market in China, regulators can use changes in energy prices to predict carbon price changes, assess the risks brought by carbon price fluctuations in advance, and put forward corresponding preventive measures. In this way, adopting appropriate risk control measures to avoid risks can improve the stability of the carbon trading market. (2) Enterprises should take full advantage of the relevant information of the energy market and control the carbon emission cost of enterprises by adjusting the energy consumption structure or improving the energy utilization rate. When the energy price changes, enterprises can predict the carbon price. If the carbon price is on the rise, enterprises should choose more clean energy or develop new technologies to improve the energy utilization rate, thus reducing the demand for carbon emissions and reducing the carbon emission cost of enterprises. (3) The government should ensure the stability of energy prices. Our research results show that the energy price can predict the carbon price well, so the stability of the energy price helps the government to set the carbon price reasonably. Specifically, the government should pay attention to improving the technical level of energy exploration and exploitation in China, increasing the domestic energy supply, and forming a more independent energy market. This can better control the source of carbon price fluctuations and fundamentally guarantee the stable and healthy development of the carbon trading market.

## Figures and Tables

**Table 1:** The trading situation of the carbon trading markets from 5 May 2014 to 22 July 2021.

Region	Abbreviation	Time Span	Trading Days
Hubei	HBEA	5 May 2014–22 July 2021	1704
Shenzhen	SZA	5 May 2014–22 July 2021	1606
Guangdong	GDEA	5 May 2014–22 July 2021	1520
Beijing	BEA	5 May 2014–22 July 2021	1093
Shanghai	SHEA	6 May 2014–22 July 2021	986
Tianjin	TJEA	5 May 2014–30 June 2021	668
Chongqing	CQEA	19 June 2014–21 July 2021	655

**Table 2:** The selection of energy prices.

Energy	Feature Name	Time Span
crude oil	Brent crude oil	5 May 2014–22 July 2021
natural gas	NYMEX natural gas	5 May 2014–22 July 2021
coal	Newcastle coal	5 May 2014–22 July 2021

**Table 3:** Two time-series data sets described.

Feature Name	Mean	Min	Max	Std	Kurtosis	Skewness
Hubei						
HBEA	23.830	10.380	53.850	6.614	0.053	0.224
crude oil	60.253	19.330	115.060	17.087	1.375	0.923
natural gas	2.830	1.550	4.790	0.630	0.770	0.696
coal	78.062	46.590	164.750	21.705	−0.090	0.667
Guangdong						
GDEA	22.537	8.100	77.000	11.154	4.471	1.853
crude oil	59.571	19.330	115.060	15.879	2.065	0.952
natural gas	2.793	1.551	4.750	0.613	1.032	0.688
coal	79.153	47.370	164.750	21.437	0.020	0.648

**Table 4:** HBEA prediction results.

Model	MAE	MSE	RMSE
SVR	2.776	9.381	3.063
MLP	1.124	1.493	1.222
RNN	0.685	1.119	1.058
LSTM	0.617	0.957	0.978

**Table 5:** GDEA prediction results.

Model	MAE	MSE	RMSE
SVR	2.756	9.119	3.020
MLP	0.653	0.791	0.889
RNN	0.527	0.427	0.653
LSTM	0.407	0.293	0.541

**Table 6:** Comparison of carbon price forecasting methods.

Paper	Time Span	Model	Accuracy (RMSE)
Hubei			
Sun and Huang [38]	2 April 2014–31 October 2019	EMD-VMD-LSTM	1.040
Wang et al. [39]	3 March 2014–3 April 2020	VMD-SE-DRNN-GRU	1.048
Our model	5 May 2014–22 July 2021	multivariate LSTM	0.978
Guangdong			
Xiong et al. [40]	1 September 2016–11 September 2018	VMD-FMRVR-MOWOA	0.570
Wang et al. [41]	20 December 2013–27 April 2020	CEEMDAN-SE-LSTM-RF	1.295
Our model	5 May 2014–22 July 2021	Multivariate LSTM	0.541

## Data Availability

The data presented in this study are available within the article.
